# Pathological periodontal pockets are associated with raised diastolic blood pressure in obese adolescents

**DOI:** 10.1186/s12903-015-0026-6

**Published:** 2015-03-24

**Authors:** Cecilia C Zeigler, Biniyam Wondimu, Claude Marcus, Thomas Modéer

**Affiliations:** Division of Pediatric Dentistry, Department of Dental Medicine, Karolinska Institutet, Box 4064, SE-141 04 Huddinge, Stockholm Sweden; National Childhood Obesity Centre, Division of Pediatrics Department of Clinical Science, Intervention and Technology, Karolinska Institutet, Stockholm, Sweden

**Keywords:** Obesity, Adolescents, Hypertension, Periodontal disease

## Abstract

**Background:**

Obesity, a well-known risk factor for developing cardiovascular disease (CVD), is associated with chronic periodontitis in adults. This cross-sectional pilot study on obese adolescents was designed to investigate whether periodontal disease in terms of pathological periodontal pockets is associated with raised blood pressure and other risk markers for CVD.

**Methods:**

The study included 75 obese subjects between 12 to 18 years of age, mean 14.5. Subjects answered a questionnaire regarding health, oral hygiene habits and sociodemographic factors. A clinical examination included Visible Plaque Index (VPI %), Gingival inflammation (BOP %) and the occurrence of pathological pockets exceeding 4 mm (PD ≥ 4 mm). Blood serum were collected and analyzed. The systolic and diastolic blood pressures were registered.

**Results:**

Adolescents with pathological periodontal pockets (PD ≥ 4 mm; n = 14) had significantly higher BOP >25% (P = 0.002), higher diastolic blood pressure (P = 0.008), higher levels of Interleukin (IL)-6 (P < 0.001), Leptin (P = 0.018), Macrophage Chemoattractant Protein-1 (MCP-1) (P = 0.049) and thyroid stimulating hormone (TSH) (P = 0.004) in blood serum compared with subjects without pathological periodontal pockets (PD ≥ 4 mm; n = 61). The bivariate linear regression analysis demonstrated that PD ≥ 4 mm (P = 0.008) and systolic blood pressure (P < 0.001) were significantly associated with the dependent variable “diastolic blood pressure”. The association between PD ≥ 4 mm and diastolic blood pressure remained significant (P = 0.006) even after adjusting for potential confounders BMI-sds, age, gender, mother’s country of birth, BOP >25%, IL-6, IL-8, Leptin, MCP-1, TSH and total cholesterol in the multiple regression analysis.

**Conclusion:**

In conclusion, this study indicates an association between pathological periodontal pockets and diastolic blood pressure in obese adolescents. The association was unaffected by other risk markers for cardiovascular events or periodontal disease. The results call for collaboration between pediatric dentists and medical physicians in preventing obesity development and its associated disorders.

## Background

Obesity, a well-known risk factor for developing cardiovascular disease (CVD), is associated with chronic periodontitis in adults and adolescents [1-4]. The main underlying pathological pathway for CVD is atherosclerosis, which arises from endothelial dysfunction driven by inflammatory processes. [5,6]. This dynamic and progressive inflammation is mediated by several inflammatory mediators including Tumor necrosis factor alpha (TNF-α), C-reactive protein (CRP), monocyte chemoattractant protein-1 (MCP-1) and IL-1β, IL-6, and IL-8 [7,8]. These inflammatory mediators are also connected with the pathogenesis and inflammatory response of periodontal disease [9-11] and they are affected in obese individuals [12]. Periodontal disease is not a localized oral lesion, but may contribute to a systemic immune response in patients [13]. The systemic inflammatory response accompanying periodontal disease has been proposed to have adverse effects on blood pressure [14]. Periodontitis may also be capable of inducing vascular inflammation, which leads to endothelial dysfunction, an initial step for CVD [14]. There are, as well, several studies presenting evidence of the oral infection theory of atherogenesis [10]. Periodontal pathogens are able to cause transient bacteremia, invading the arterial wall and possibly lead to vascular inflammation and atherosclerosis [14]. Interestingly, periodontal therapy has been demonstrated, in clinical interventions studies, to decrease levels of the biomarkers CRP and IL-6 in serum as well as improving endothelial function [14,15]. In addition, periodontal treatment has been shown to reduce levels of cholesterol and triglycerides both in adults [16,17] and children [18]. There is also emerging evidence that successful periodontal treatment might help reduce blood pressure in patients with hypertension [19]. As raised blood pressure at a young age is a strong predictor for cardiovascular mortality [20,21], it is interesting to test the hypothesis whether there is any association between blood pressure and early signs of periodontitis in adolescents. This cross-sectional pilot study was therefore designed to investigate whether the occurrence of pathological periodontal pockets might be associated with raised blood pressure or other risk markers for CVD in obese adolescents.

## Methods

The present cross-sectional pilot study included 84 obese subjects who were recently referred to the National Childhood Obesity Center, Karolinska University Hospital, Huddinge, from their primary care giver or school nurse. Subjects were consecutively referred to the Division of Pediatric Dentistry, Karolinska Institutet for oral examination as part of the medical investigation at the National Childhood Obesity Center. Approximately one subject per week underwent the registration procedure and this study was conducted during an 18 month period during 2006 and 2007. All subjects lived within the hospitals catchment area and traveled less than one hour to get to the Center. Body weight (kg) and height (m) were determined and obesity was expressed as Body Mass Index (BMI) (kg/m^2^) as well as by BMI adjusted for age and gender (BMI-sds) [22]. The following exclusion criteria were used: under 12 years of age, any antibiotic treatment during the last 3 months, ongoing orthodontic treatment and/or subjects admitting to smoking. After excluding subjects that did not meet the criteria, 78 subjects remained. A further 3 subjects were removed as sample sets were not complete. Finally 75 subjects remained (Table 1).

**Table 1:** **Characteristics and oral hygiene habits of the subjects with or without pathological periodontal pockets (PD ≥ 4 mm)**

	**With PD ≥4 mm**	**Without PD ≥4 mm**	
**Variables**	**(n = 14)**	**(n = 61)**	**P-value** ^**1**^
Male/female ratio	8/6	34/27	0.925
	**Mean (SD)**	**Mean (SD)**	
Age	14.5 (1.6)	14.5 (1.6)	0.998
Weight (kg)	105.0 (21.8)	108.5 (23.4)	0.855
Height (m)	1.71 (0.12)	1.70 (0.10)	0.637
BMI-sds^2^	5.3 (1.3)	5.7 (1.1)	0.209
	**n (%)**	**n (%)**	
Chronic disease	3 (21)	17 (28)	0.629
Diabetes type 2	1 (7)	5 (8)	0.885
Thyroid dysfunctions	1 (7)	4 (7)	0.938
Daily use of prescribed medication	3 (21)	21 (34)	0.354
Parents Country of birth			
Mother - Sweden	7 (50)	35 (57)	
- Not Sweden	7 (50)	26 (43)	0.497
Father - Sweden	6 (43)	35 (57)	
- Not Sweden	8 (57)	26 (43)	0.260
Parents education^3^			
Both parents ≤12år	11 (79)	40 (66)	
One parent >12år	1 (7)	14 (23)	
Both parents >12år	1 (7)	7 (11)	0.277
Tooth brushing			
Daily morning	7 (50)	30 (49)	0.832
Daily evening	6 (43)	32 (52)	0.450
Daily flossing	1 (7)	2 (3)	0.948

^1^ONEWAY ANOVA and Chi^2^ used as statistical methods.

^2^According to Rolland-Cacherra [22].

^3^Education level of one parent was unknown to the participating subject.

We certify that all applicable institutional and governmental regulations concerning the ethical use of human volunteers were followed during the research. The Ethics Committee at Karolinska University Hospital Huddinge approved the study and all subjects and parents gave consent before participating. All subjects co-operated satisfactorily during examinations, measurement and sample-taking situations.

### Questionnaire

All adolescents answered a questionnaire that covered topics of their medical condition, medication, meal frequency, oral hygiene habits, smoking habits, as well as their parents’ education and country of birth. Parents’ country of birth was categorized into “born in Sweden” or “born abroad”. The educational level of the parents was stratified according to the years of schooling as either ≤ 12 or > 12 years. Participants under 18 years were assisted by a parent when answering the questionnaire.

### Clinical examination

Dental plaque on tooth surfaces was recorded when clearly visible and expressed using the Visible Plaque Index (VPI) [23]. Gingival inflammation was based on Bleeding on Probing (BOP) [24]. Both VPI and BOP was recorded at six sites on each tooth (wisdom teeth excluded). The proportion of surfaces (%) with visible dental plaque and gingival inflammation, respectively, was calculated for each subject.

Pocket depth (mm) was recorded by using a graded periodontal probe (LM-instruments OY, Parainen, Finland) and measured to the nearest mm. The occurrence of pathological periodontal pockets was classified when the subject exhibited one or more sites with a pocket depth of ≥ 4 mm. Erupting and deciduous teeth were excluded.

Supragingival calculus was recorded on all teeth as present when clearly visible. Subgingival calculus was recorded on the radiographs taken as well as clinically after probing the gingival sulcus. The oral clinical recording was performed by one of the authors (CZ).

Systolic and diastolic blood pressure was measured in the sitting position with a mercury sphygmomanometer with a standard pressure cuff of the correct size on the left arm. Blood pressures were measured in duplicates. A third measurement was taken if the two readings differed more than 5 mm Hg and the mean of the two closest readings was used. The same experienced nurse practitioner performed all measurements.

Blood samples were taken after an overnight fast starting at midnight. Fasting was confirmed by the child and parent before collecting blood. Blood samples for High density lipoprotein (HDL) were analyzed immediately. Remaining samples were kept frozen in-80°C until analyzed. HDL, TSH and CRP-sensitivity analyzes were performed at a certified laboratory (Department of Clinical Chemistry, Karolinska University Hospital). IL-1β, IL-6, IL-8, MCP-1 and Leptin were analyzed using commercially available Luminex kits (Linco Research, Inc., Missouri, USA). TNF-α levels were measured using Bio-Plex cytokine assay (Bio-Rad laboratories, California, USA).

### Statistics

For analyzing the data, frequency tables, cross tables, ONEWAY ANOVA, Chi-square, linear regression were used by SPSS version 21.0 (SPSS, Chicago, IL, USA). Bivariate analyses were carried out between the dependent variable diastolic blood pressure or systolic blood pressure and the independent variables by applying a linear regression model. In the multiple linear regression analysis with diastolic blood pressure as dependent variable, the independent variable PD ≥ 4 mm was adjusted for potential confounders (BMI-sds, age, gender, mother’s country of birth, BOP >25%, IL-6, IL-8, Leptin, MCP-1, TSH and total cholesterol).

Confidence Interval 95% was calculated. Level of significance was accepted at P-values less than 0.05. In case of biochemical variables, the Bonferroni test adjusted for intra correlations between the biomedical variables and a P-value of <0.01 was required to declare significance to avoid chance of mass significance.

## Results

Of the subjects included in this study (n = 75), 14 displayed pathological periodontal pockets (PD ≥ 4 mm) and 61 were without pathological periodontal pockets (PD < 4 mm, n = 61). There was no significant difference between adolescents with and without PD ≥ 4 mm in regards to age, gender, BMI-sds, tooth brushing habits, sociodemographic factors, medical history or visible plaque index (VPI > 25%) (Table 1). Adolescents with PD ≥ 4 mm had significantly higher presence of BOP > 25%, higher diastolic blood pressure (P = 0.008) and significantly higher levels of IL-6 (P < 0.001), Leptin (P = 0.018), MCP-1 (P = 0.049) and TSH (P = 0.004) in blood serum (Table 2). Presence of calculus, IL-1β, IL-8, TNF-α, total cholesterol, LDL/HDL quotient, Hba1c, or CRP levels did not differ significantly between the two groups and neither did systolic blood pressure (Table 2). Other blood markers were tested (Adiponectin, PAI-1, Insulin, Glucose, Alat, Creatinine, Apolipo A1, Apolipo B, fP-TG, Urea, IGF-1) and no significant differences were demonstrated between the groups (data not shown).

**Table 2:** **Clinical and biomedical variables in blood serum of the subjects with or without pathological periodontal pockets (PD ≥4 mm)**

	**With PD ≥4 mm**	**Without PD ≥4 mm**	
	**(n = 14)**	**(n = 61)**	
**Variables**	**Number (%)**	**Number (%)**	**P-value** ^**1**^
Presence of dental calculus			
Supragingival	8 (57)	22 (36)	0.151
Subgingival	4 (29)	10 (16)	0.586
VPI			
0-25%	8(57)	47 (77)	
> 25%	6 (43)	14 (23)	0.132
BOP			
0-25%	5 (36)	47 (77)	
> 25%	9 (64)	1 (23)	0.002
	**Mean (SD)**	**Mean (SD)**	
Adiponectin (μg/ml)	13.5 (74.3)	12.3 (802)	0.641
PAI-1 Tot (ng/ml)	42.8 (19.2)	944 (6635)	0.642
IL-1β (pg/ml)	0.8 (0.8)	1.1 (4.1)	0.819
IL-6 (pg/ml)	37.5 (44.9)	10.5 (11.3)	<0.001
IL-8 (pg/ml)	15.2 (7.7)	33.9 (106.8)	0.548
Leptin (ng/ml)	66.3 (54,1)	41.2 (23.0)	0.018
TNF-α (pg/ml)	6.9 (2.5)	6.8 (3.1)	0.848
MCP-1 (pg/ml)	256 (104)	464 (352)	0.049
Total cholesterol (mmol/l)	3.9 (0.7)	4.0 (0.7)	0.645
LDL/HDL quotient	2.1 (1.0)	2.2 (0.7)	0.404
Hba1c (mmol/mol)	4.5 (0.4)	4.6 (0.4)	0.182
CRP (mg/l)	2.6 (2.7)	4.7 (4.1)	0.071
TSH (mlU/l)	3.9 (3.0)	2.5 (1.1)	0.004
Blood pressure (mmHg)			
-Systolic	119 (9)	116 (9)	0.294
-Diastolic	74 (5)	67 (7)	0.008
Insulin resistance Bergman			
-Si (×10^−4^/min/pM)	1.9 (1.3)	1.5 (1.4)	0.313
-Air (pM)	1930 (1782)	3107 (3692)	0.254

^1^ONEWAY ANOVA as statistical method.

Subjects with PD ≥ 4 mm had an average diastolic blood pressure of 74 mmHg whilst subjects without PD ≥ 4 mm had average diastolic blood pressure of 67 mmHg.

In a bivariate linear regression analysis with diastolic blood pressure as dependent variable, following variables were significantly associated: PD ≥ 4 mm (P = 0.008) and systolic blood pressure (P < 0.001) (Table 3). As demonstrated in Table 4, PD ≥ 4 mm remained significantly (P = 0.006) associated with diastolic blood pressure even after adjusting for BMI-sds, age, gender, mother’s country of birth, BOP >25%, IL-6, IL-8, Leptin, MCP-1, TSH and total cholesterol in a multiple linear regression analysis.

**Table 3:** **Bivariate linear regression analysis with diastolic blood pressure as dependent variable**

**Variable**	**β-coefficient**	**Std. error**	**P**	**95% CI**
Age	0.09	0.59	0.481	−0.76 – 1.60
Gender	−0.13	1.87	0.303	−5.66 – 1.80
Mother country of birth (not Sweden)	0.42	3.16	0.056	−0.15 – 12.48
Father country of birth (not Sweden)	−0.34	3.16	0.116	−11.37 – 1.29
BMI-sds	0.07	0.85	0.593	−1.25 – 2.15
PD ≥4 mm	0.33	0.98	0.008	1.69 – 10-69
Presence of dental calculus				
Supragingival	−0.02	1.92	0.901	−4.07 – 3.59
Subgingival	0.05	1.17	0.669	−1.8 – 2.84
VPI (>25)%	0.02	2.11	0.861	−3.85 – 4.59
BOP (>25%)	0.06	2.07	0.662	−3.23 – 5.05
IL-1β (pg/ml)	−0.16	0.29	0.301	−0.89 – 0.28
IL-6 (pg/ml)	−0.01	0.04	0.993	−0.08 – 0.09
IL-8 (pg/ml)	−0.25	0.01	0.072	−0.04 – 0.01
Leptin (ng/ml)	0.08	0.01	0.581	−0.01 – 0.01
TNF-α (pg/ml)	0.04	0.33	0.793	−0.58 – 0.75
MCP-1 (pg/ml)	−0.09	0.01	0.491	−0.01 – 0.01
Total cholesterol (mmol/l)	−0.23	1.25	0.065	−4.87 – 0.15
LDL/HDL quotient	−0.10	1.42	0.460	−3.90 – 1.79
Hba1c (mmol/mol)	−0.03	2.15	0.823	−4.78 – 3.81
CRP (mg/l)	−0.012	0.23	0.924	−0.49 – 0.45
TSH (mlU/l)	−0.01	0.54	0.960	−1.10 – 1.05
Systolic blood pressure (mmHg)	0.44	0.09	<0.001	0.16 – 0.53

**Table 4:** **Multiple linear regression analysis with diastolic blood pressure as dependent variable**

**Variable**	**β-coefficient**	**Std. error**	**P**	**95% CI**
PD ≥ 4 mm-unadjusted	0.33	0.98	0.008	1.69 – 10-69
-Adjusted for				
BMI-sds	0.36	2.29	0.005	11.29 – 2.11
Age	0.33	2.26	0.008	1.69 – 10.73
Gender	0.33	2.26	0.009	1.59 – 10.61
Mother’s country of birth	0.32	2.38	0.013	1.36 – 10.87
BOP >25%	0.35	2.39	0.008	1.74 – 11.31
Leptin (ng/ml)	0.43	2.60	0.005	2.48 – 12.93
MCP-1 (pg/ml)	0.37	2.53	0.009	1.85 – 11.99
TSH (mlU/l)	0.37	2.37	0.005	2.17 – 11.67
IL-6 (pg/ml)	0.46	2.77	0.002	3.49 – 14.60
IL-8 (pg/ml)	0.35	2.41	0.008	0.81 – 11.47
Total cholesterol (mmol/l)	0.31	2.23	0.012	1.31 – 10.25
PD ≥ 4 mm adjusted for BMI-sds, age, gender, mother’s country of birth, BOP > 25%, leptin, MCP-1, TSH, IL-6, IL-8 and cholesterol	0.48	3.18	0.007	2.68 – 15.67

In a bivariate linear regression analysis with systolic blood pressure as dependent variable, diastolic blood pressure (P > 0.001) as well as serum level of TSH (P = 0.039) showed significant association (data not shown).

## Discussion

The main finding in this study demonstrates an association between periodontal disease in terms of pathological periodontal pockets and diastolic blood pressure in obese adolescents. Obese subjects with pathological periodontal pockets had significantly higher diastolic blood pressure (74 mmHg) compared with periodontally healthy obese adolescents (67 mmHg). Pediatric subjects with obesity have been reported to have higher systolic and diastolic blood pressure than their normal weight counterparts [24,25]. The average diastolic blood pressure for obese adolescents is reported to vary from 62 mmHg to 70 mmHg [26,27] with a frequency of prehypertension of approximately 20% [28,29]. However, there is still an on-going debate on how and when to diagnose children and adolescents as pre-hypertensive, hypertensive or normal [30-32]. Considering this lack of agreement and the limited number of participants included in this study we used blood pressure as a continuous variable in the statistical analysis rather than dividing the subjects into groups based on level of blood pressure. Consequently, we report our findings as raised diastolic blood pressure, instead of prehypertension or pathological hypertension.

In the current study we used one-time blood pressure measurement in a resting position which is an acceptable screening method [33]. However, most recommend that to diagnose a child or adolescent with hypertension, a 24 hour evaluation is necessary. [34,35]. Based on the blood pressure method used, one has to consider that other stress factors such as travel time and transportation method might have affected blood pressure levels.

The endpoint of inflammatory periodontal disease includes alveolar bone resorption and subsequently loss of teeth. Subjects in this study are young and the effect of chronic inflammation on periodontal tissue needs longer exposure time until alveolar bone loss is possible to detect on the radiographs. Therefore, we used pathological periodontal pocket (PD ≥ 4 mm), a variable, commonly used as a periodontal risk indicator in young subjects [1] in the statistical analysis. To investigate if indeed the systemic inflammatory response accompanying periodontal disease could be part of the mechanism of atherosclerosis and its cardiovascular ill-effects [36,37], we used diastolic blood pressure as the dependent variable in the linear regression model. Of the variables studied, only PD ≥4 mm and systolic blood pressure demonstrated a significant association in the bivariate linear regression model (Table 3). We further tested the association between diastolic blood pressure and PD ≥4 mm in a multiple linear regression model (Table 4). Variables known to have effect on blood pressure in adolescents: BMI-sds, gender and age [34] were entered into the model. Since mother’s country of birth, IL-8 and Cholesterol were near significant in the bivariate analysis these variables were, as well, entered into the model. The cytokine IL-8 is, as well, known to be involved in the pathogenesis of hypertension [8]. As shown in Table 2 BOP >25%, IL-6, Leptin, TSH and MCP-1 varied significantly between the subjects with or without PD ≥4 mm, therefor they were, as well, entered into the model. The association between PD ≥4 mm and diastolic blood pressure was not affected by any of the potential confounders entered into the model (Table 4). The significant higher level of IL-6, TSH and Leptin in serum in the subjects with PD ≥4 mm (Table 2) further support the notion that, in obese subjects, periodontal disease is part of an systemic inflammation that might, in the long run, lead to atherosclerosis and CVD [38,39]. In addition, it has recently been reported that an increase in leptin levels in diet-induced obese rodents drives an increase in blood pressure, an effect that was not seen in animals deficient of leptin or leptin receptors [40]. Further support comes from clinical studies demonstrating that periodontal treatment lowers the levels of both IL-6 and Leptin in serum [41] and thereby decreases the burden of systemic inflammation.

TSH has not previously been connected to periodontal disease, but is reported to be enhanced in serum in obese subjects [42]. In this study, the subjects without PD ≥4 mm had TSH levels corresponding to average levels in healthy individuals, whilst the subjects with PD ≥4 mm had relatively higher levels. Interestingly, it has been reported that increases in TSH levels are associated with increases in both systolic and diastolic blood pressure as well as other cardiovascular risk factors [43-45].

Our finding that there is an association between PD ≥4 mm and diastolic blood pressure is compatible with the finding in a similar age group that overweight/obese adolescents had significantly higher blood pressure and more gingival inflammation than their normal weight counterparts [24]. However, the authors did not report if there was a direct association between periodontal inflammation and blood pressure. In addition, our finding regarding the association between PD ≥4 mm and raised diastolic blood pressure is well compatible with the view that adolescents positive for 3 or more variables, associated with metabolic syndrome, are more likely to display gingival inflammation than healthy individuals [46]. Interestingly, the current epidemiological data also indicate that there is an association between periodontal disease and hypertension in adults as well [14,47].

Perpending the finding, that diastolic blood pressure at 18 years of age seems to be a stronger predictor for cardiovascular mortality than systolic blood pressure [20], it is interesting to consider whether the occurrence of PD ≥4 mm in children with obesity might be an early marker for future cardiovascular events.

This pilot study has, however, several limitations. Since no normal weight participants are included, the association between periodontal disease and raised diastolic blood pressure might be specific for the obese condition. We did not register the duration of obesity of the subjects which probably also, in addition to the severity of obesity, is of importance when looking at the association between periodontal risk factors and blood pressure. Since this study has a cross sectional design, a longitudinal study is needed before any conclusions can be drawn.

## Conclusion

This study indicates an association between pathological periodontal pockets and diastolic blood pressure in obese adolescents. The association was unaffected by other risk markers for cardiovascular events or periodontal disease. The results call for collaboration between pediatric dentists and medical physicians in preventing obesity development and its associated disorders.

## Abbreviations

BMI-sds: Body mass index standard deviation score
BOP: Bleeding on probing
CRP: C-reactive protein
CVD: Cardiovascular disease
fP-TG: Fasting plasma triglycerides
Hba1c: Glycated hemoglobin
HDL: High density lipoprotein
IGF-1: Insulin-like growth factor 1
IL: Interleukin
LDL: Low density lipoprotein
MCP-1: Macrophage chemoattractant protein-1
PAI-1: Plasminogen activator inhibitor −1
PD ≥ 4 mm: Gingival pathological pockets ≥ 4 mm
TNF-α: Tumor necrosis factor alpha
TSH: Thyroid stimulating hormone
VPI: Visible plaque index

## References

[1] Modeer T, Blomberg C, Wondimu B, Lindberg TY, Marcus C (2011). Association between obesity and periodontal risk indicators in adolescents. Int J Pediatr Obes.

[2] Saito T, Shimazaki Y, Sakamoto M (1998). Obesity and periodontitis. N Engl J Med.

[3] Suvan J, D'Aiuto F, Moles DR, Petrie A, Donos N (2011). Association between overweight/obesity and periodontitis in adults. A systematic review. Obes Rev.

[4] Chaffee BW, Weston SJ (2010). Association between chronic periodontal disease and obesity: a systematic review and meta-analysis. J Periodontol.

[5] Libby P (2002). Inflammation in atherosclerosis. Nature.

[6] Brevetti G, Schiano V, Chiariello M (2008). Endothelial dysfunction: a key to the pathophysiology and natural history of peripheral arterial disease?. Atherosclerosis.

[7] Dervaux N, Wubuli M, Megnien JL, Chironi G, Simon A (2008). Comparative associations of adiposity measures with cardiometabolic risk burden in asymptomatic subjects. Atherosclerosis.

[8] Martynowicz H, Janus A, Nowacki D, Mazur G (2014). The role of chemokines in hypertension. Adv Clin Exp Med.

[9] Yucel-Lindberg T, Bage T (2013). Inflammatory mediators in the pathogenesis of periodontitis. Expert Rev Mol Med.

[10] Teles R, Wang CY (2011). Mechanisms involved in the association between periodontal diseases and cardiovascular disease. Oral Dis.

[11] Gupta M, Chaturvedi R, Jain A (2013). Role of monocyte chemoattractant protein-1 (MCP-1) as an immune-diagnostic biomarker in the pathogenesis of chronic periodontal disease. Cytokine.

[12] Ahima RS, Osei SY (2008). Adipokines in obesity. Front Horm Res.

[13] Gomes MS, Blattner TC, Sant'Ana Filho M, Grecca FS, Hugo FN, Fouad AF (2013). Can apical periodontitis modify systemic levels of inflammatory markers? A systematic review and meta-analysis. J Endod.

[14] Leong XF, Ng CY, Badiah B, Das S (2014). Association between Hypertension and Periodontitis: Possible Mechanisms. Sci World J.

[15] Higashi Y, Goto C, Hidaka T, Soga J, Nakamura S, Fujii Y (2009). Oral infection-inflammatory pathway, periodontitis, is a risk factor for endothelial dysfunction in patients with coronary artery disease. Atherosclerosis.

[16] Caula AL, Lira-Junior R, Tinoco EM, Fischer RG (2014). The effect of periodontal therapy on cardiovascular risk markers: a 6-month randomized clinical trial. J Clin Periodontol.

[17] D'Aiuto F, Parkar M, Nibali L, Suvan J, Lessem J, Tonetti MS (2006). Periodontal infections cause changes in traditional and novel cardiovascular risk factors: results from a randomized controlled clinical trial. Am Heart J.

[18] Bresolin AC, Pronsatti MM, Pasqualotto LN, Nassar PO, Jorge AS, da Silva EA (2014). Effectiveness of periodontal treatment on the improvement of inflammatory markers in children. Arch Oral Biol.

[19] Vidal F, Cordovil I, Figueredo CM, Fischer RG (2013). Non-surgical periodontal treatment reduces cardiovascular risk in refractory hypertensive patients: a pilot study. J Clin Periodontol.

[20] Sundstrom J, Neovius M, Tynelius P, Rasmussen F (2011). Association of blood pressure in late adolescence with subsequent mortality: cohort study of Swedish male conscripts. BMJ.

[21] Mancia G, Laurent S, Agabiti-Rosei E, Ambrosioni E, Burnier M, Caulfield MJ (2009). Reappraisal of European guidelines on hypertension management: a European Society of Hypertension Task Force document. Blood Press.

[22] Rolland-Cachera MF, Sempe M, Guilloud-Bataille M, Patois E, Pequignot-Guggenbuhl F, Fautrad V (1982). Adiposity indices in children. Am J Clin Nutr.

[23] Ainamo J, Bay I (1975). Problems and proposals for recording gingivitis and plaque. Int Dent J.

[24] Franchini R, Petri A, Migliario M, Rimondini L (2011). Poor oral hygiene and gingivitis are associated with obesity and overweight status in paediatric subjects. J Clin Periodontol.

[25] Lurbe E, Alvarez V, Liao Y, Tacons J, Cooper R, Cremades B (1998). The impact of obesity and body fat distribution on ambulatory blood pressure in children and adolescents. Am J Hypertens.

[26] Polderman J, Gurgel RQ, Barreto-Filho JA, Roelofs R, Ramos RE, de Munter JS (2011). Blood pressure and BMI in adolescents in Aracaju, Brazil. Public Health Nutr.

[27] Camhi SM, Katzmarzyk PT (2011). Prevalence of cardiometabolic risk factor clustering and body mass index in adolescents. J Pediatr.

[28] Cao ZQ, Zhu L, Zhang T, Wu L, Wang Y (2012). Blood pressure and obesity among adolescents: a school-based population study in China. Am J Hypertens.

[29] McNiece KL, Poffenbarger TS, Turner JL, Franco KD, Sorof JM, Portman RJ (2007). Prevalence of hypertension and pre-hypertension among adolescents. J Pediatr.

[30] Mourato FA, Lima Filho JL, Mattos SD. Comparison of different screening methods for blood pressure disorders in children and adolescents. J Pediatria. 2014. doi:10.1016/j.jped.2014.08.008. 10.1016/j.jped.2014.08.008 25475553

[31] Xi B, Zhang M, Zhang T, Li S, Steffen LM (2015). Simplification of childhood hypertension definition using blood pressure to height ratio among US youths aged 8-17years, NHANES 1999–2012. Int J Cardiol.

[32] Flynn JT, Daniels SR, Hayman LL, Maahs DM, McCrindle BW, Mitsnefes M (2014). Update: ambulatory blood pressure monitoring in children and adolescents: a scientific statement from the American Heart Association. Hypertension.

[33] Buchanan S, Orris P, Karliner J (2011). Alternatives to the mercury sphygmomanometer. J Public Health Policy.

[34] National High Blood Pressure Education Program Working Group on High Blood Pressure in C, Adolescents (2004). The fourth report on the diagnosis, evaluation, and treatment of high blood pressure in children and adolescents. Pediatrics.

[35] Westerstahl M, Marcus C (2010). Association between nocturnal blood pressure dipping and insulin metabolism in obese adolescents. Int J Obes (Lond).

[36] Nakajima T, Yamazaki K (2009). Periodontal disease and risk of atherosclerotic coronary heart disease. Odontology.

[37] Tonetti MS, Van Dyke TE, working group 1 of the joint EFPAAPw (2013). Periodontitis and atherosclerotic cardiovascular disease: consensus report of the Joint EFP/AAP Workshop on Periodontitis and Systemic Diseases. J Periodontol.

[38] Gundala R, Vk C, Ramalingam K (2012). Association of Leptin in Periodontitis and Acute Myocardial Infarction. J Periodontol.

[39] Cochran DL (2008). Inflammation and bone loss in periodontal disease. J Periodontol.

[40] Simonds SE, Pryor JT, Ravussin E, Greenway FL, Dileone R, Allen AM (2014). Leptin mediates the increase in blood pressure associated with obesity. Cell.

[41] Shimada Y, Komatsu Y, Ikezawa-Suzuki I, Tai H, Sugita N, Yoshie H (2010). The effect of periodontal treatment on serum leptin, interleukin-6, and C-reactive protein. J Periodontol.

[42] Nannipieri M, Cecchetti F, Anselmino M, Camastra S, Niccolini P, Lamacchia M (2009). Expression of thyrotropin and thyroid hormone receptors in adipose tissue of patients with morbid obesity and/or type 2 diabetes: effects of weight loss. Int J Obes (Lond).

[43] Asvold BO, Bjoro T, Vatten LJ (2013). Associations of TSH levels within the reference range with future blood pressure and lipid concentrations: 11-year follow-up of the HUNT study. Eur J Endocrinol.

[44] Klein I, Danzi S (2007). Thyroid disease and the heart. Circulation.

[45] Weiss IA, Bloomgarden N, Frishman WH (2011). Subclinical hypothyroidism and cardiovascular risk: recommendations for treatment. Cardiol Rev.

[46] Lee KS, Lee SG, Kim EK, Jin HJ, Im SU, Lee HK (2015). Metabolic Syndrome Parameters in adolescents may be determinants for the future periodontal diseases. J Clin Periodontol.

[47] Tsakos G, Sabbah W, Hingorani AD, Netuveli G, Donos N, Watt RG (2010). Is periodontal inflammation associated with raised blood pressure? Evidence from a National US survey. J Hypertens.

